# How Much Energy Vaquejada Horses Spend in a Field Simulation Test?

**DOI:** 10.3390/ani11123421

**Published:** 2021-11-30

**Authors:** Clarisse S. Coelho, Ticiane D. R. P. Sodre, Lara N. Sousa, Renata F. Siqueira, Helio C. Manso Filho, Francesca Aragona, Francesco Fazio

**Affiliations:** 1 Faculdade de Medicina Veterinária, Universidade Lusófona de Humanidades e Tecnologias (ULHT), Campo Grande 376, 1749-024 Lisboa, Portugal; claricoelho09@gmail.com; 2 Escola de Medicina Veterinária, Universidade Federal da Bahia (UFBA), Salvador 40170-110, Brazil; ticy_sodre@hotmail.com (T.D.R.P.S.); laranunesmvet@gmail.com (L.N.S.); 3 Faculdade de Medicina Veterinária, Universidade Federal de Santa Maria (UFSM), Santa Maria 97105-900, Brazil; refarinelli@yahoo.com.br; 4 Núcleo de Pesquisa Equina, Universidade Federal Rural de Pernambuco (UFRPE), Recife 52171-900, Brazil; equideo@gmail.com; 5 Department of Veterinary Sciences, University of Messina, Polo Universitario Annunziata, 98168 Messina, Italy; franceca.17@gmail.com

**Keywords:** energy expenditure, equine, equestrian performance, field tests

## Abstract

**Simple Summary:**

Vaquejada is an important Brazilian equine discipline characterized by high-intensity and short-duration exercise that should influence the energetic cost for athletic horses. The aim of this study was to analyse this effect by a vaquejada simulation test (VST), in order to evaluate energy expenditure (EE), transport cost (COT) and metabolic energy requirement (Pmet) in horses. Eight Quarter Horses were evaluated in a VST composed of three races (130–150 m each) with a 5-min interval between them. All horses used an integrated heart rate (HR) and GPS monitoring system (V800, Polar Electro, Lake Success, NY, USA) to calculate the energetic index (EE, COT and Pmet). Furthermore, blood samples were collected for lactate analysis at rest, immediately after the first, second and third race and after 30 min of recovery. The results highlight that pull horses (PH) had higher EE and COT, while helper horses (HH) had higher Pmet. Although practicing the same sport, PH and EE must be considered different athletes by veterinarians, owners and all practitioners of this specific sport.

**Abstract:**

Vaquejada is a high-intensity and short-duration exercise in which helper horses (HH) are responsible to keep a bull running in a line while pull horses (PH) work to put the bull down after 100 m of running. The purpose of this study was to quantify and compare energy expenditures (EE), transport costs (COT) and metabolic energy requirements (Pmet) of horses used in Vaquejada. Thus, eight Quarter Horses, in randomly formed pairs, performed a vaquejada simulation test (VST), which consisted of three races on a sand track (130–150 m), with a 5-min interval between them. All horses used an integrated heart rate (HR) and GPS monitoring system (V800, Polar Electro) and, from these data, EE, COT and Pmet were calculated using the formulas: EE (J/kg/min) = 0.0566 × HR^1.9955^, COT = (HR-35)/kg/m × 10^3^ and Pmet = (HR-35)/min/kg. Blood samples were collected for lactate analysis at rest, immediately after the first, second and third race and after 30 min of recovery. Data obtained were submitted to one-way ANOVA and Tukey tests (*p* ≤ 0.05). In VST, HH had higher EE and higher HR at trot; while PH presented higher EE and HR at canter. Finally, considering total VST, PH had higher EE and COT, while HH had higher Pmet. Lactate was higher in PH. Despite practicing the same sport, PH and HH should be considered distinct athletes, and these must be considered to set up appropriate physical and nutritional programs, which will lead to better performance and guarantees of well-being.

## 1. Introduction

The direct measurement of oxygen consumption (VO_2_) in a field evaluation of athletic horses is impractical [1]. However, VO_2_ has a linear mathematical relation with heart rate (HR) and velocity [2,3,4,5], which makes HR a useful tool for estimating energy metabolism during locomotion [6], especially in constant velocity and submaximal intensity exercises [3,7,8]. In addition, measuring HR in field tests is easier, accessible and less costly [1,9] and has a huge advantage when considering genuine training conditions, such as rider load, behaviour and type of surface [9,10]. Portable systems, adapted for human applications [11], has enabled the indirect measurement of VO_2_ in a wide variety of equestrian activities [1,5,9,10,12,13]. Another important field metabolic measurement, includes measuring blood lactate [10,14], which helps estimate the aerobic and anaerobic contributions to energy expenditure [8]. However, the estimation of energy expenditure in equestrian sports must include moments of acceleration and deceleration, even at lower speeds, which represent essential elements in sports, such as show jumping, dressage and vaquejada, in horses and, similarly, soccer, in humans [11]. Recently, HRs have been used to quantify the cost of transport (COT), and metabolic power (Pmet), defining energy expenditure (EE) in horses used in different equestrian sports [12,13]. Vaquejada is considered a cultural sport of the northeast region of Brazil, in which two Quarter Horses work together to pull a bull down after a running for ~15 s in ~100 m [15,16]. As it is considered a high-intensity and short-duration exercise [17,18], energy production occurs mainly by anaerobic pathways [19]. Horses used in this particular equine discipline play different roles during the physical effort [20,21]. Pull horses are responsible to put the bull down; meanwhile, helper horses are responsible to run along with the bull, aligning it with the pull horse [21]. Previous studies have already shown that vaquejada horses spend more energy than those used for riding or marcha (four-beat stepping gait) [5]. However, differences between both athletes must be well documented to properly manage and underpin training and nutritional programs of those animals. The hypothesis of the authors is that pull horses execute a more intense effort, while helper horses perform a more aerobic exercise. Therefore, the objective of this present study was to quantify and compare energy expenditure (EE), cost of transport (COT) and metabolic power (Pmet) of pull and helper horses in vaquejada simulation tests.

## 2. Materials and Methods

This project was approved by the Ethics, Bioethics and Animal Welfare Committee at the Federal University of Bahia (CEUA-UFBA-BA, number 08/2018).

Animals: For this present research, eight Quarter Horses were used, with an average weight of 428 kg and average age of 8.8 years old. Animals were selected according to previous clinical evaluation and regular haematology and biochemical analysis at rest conditions, to ensure their healthy status (data not shown). All animals belonged to equine training centres at Camaçari-BA (12°41′47″ S, 38°19′24″ O) and Dom Macedo Costa-BA (12°58′35″ S, 39°8′56″ O). The horses underwent a same food and health management with diet consisting of Transvala (*Digitaria decumbens*) and/or Tifton (*Cynodon dactylon* vr. Dactylon) hay *ad libitum*, and commercial concentrate (with 12% crude protein; 1.5 kg/100 kg body weight), divided in two (5 a.m. and 5 p.m.) or three (5 a.m., 11 a.m., 5 p.m.) daily portions, according to competition season. Water was always available, and an inorganic mineral salt mixture was offered twice a week. All horses had been exercising regularly and they had been training for vaquejada practice for at least 6 months. Briefly, the training program performed in both properties was analogous and consisted of 40 min of walking alternating with galloping, 3–4 times per week; 4–6 racings with bulls (covering the distance usually executed in official events but without putting the bull down), 2–3 times per week; rest or competitions during the weekends. During training periods, each helper horse worked with two pull horses, so they worked twice as much as pull horses. Prior to competitions, the intensity of training was usually increased. Generally, all faults presented by the horses during training and/or competitions were individually corrected and, mainly, they could occur in three specific moments: the opening of the corral gate for the bull, the race and at the area for the bull’s fall.

Vaquejada Simulation Tests (VST): VST were performed in accordance with previous protocols [16,18] and also with the Associação Brasileira de Vaquejada regulation (Brazilian Association of Vaquejada-ABVAQ). For the protocol, a bull was released from a chute to two awaiting rider/horse pairs, one designated the pull horse (PH), the other the helper horse (HH). HH galloped, keeping the bull running in line down the field so that the rider on the PH could firmly take hold of the bull’s tail to bring the animal down in a soft sand track (130–150 m) after 100 m of running. Each pair of horses, which were randomly selected, ran three times (one vaquejada cycle), with a 5-min rest between races. Bulls ran only one time. All the physical trials were performed in the morning, between 7 a.m. and 11 a.m., between February and March (summer season in the Southern Hemisphere) with a local mean temperature of 30 °C and mean relative humidity of ~72%, typical of tropical regions. Sand ground was dry, and all animals were ridden by their usual and experienced riders, according to welfare precepts from Coelho et al. [22]. Mean weight of riders was 75 kg for pull horses riders and 72.5 kg for helper horses riders; weight of leather Polo saddle (adapted) and accessories was estimated in ~10 kg.

Heart rate and speed monitoring: During the VST, all horses used an integrated heart rate (HR) and GPS monitoring system (V800, Polar Electro, Lake Success, NY, USA) which recorded HR, speed and distance every 1 s throughout the entire duration of exercise. Later, data were transferred and analysed using Polar Flow Software (Polar Electro, Lake Success, NY, USA). This equipment has been previously used for horses [16,23].

Determination of energy expenditure: Determination of energy expenditure (EE), net cost transport (COT) and metabolic power (Pmet) were calculated using formulas used in previous studies [12,13], considering mean HR (beats/min) of each pace (walk, trot and gallop), total body weight (kg; considering horse, riders, and accessories), and distance run (m) for COT and time (min) for Pmet.

EE (J kg^−1^ min^−1^) = 0.0566 × HR^1.9955^ [24]

COT (beats kg^−1^ m^−1^ × 10^3^) = (HR − 35) kg^−1^ m^−1^ × 10^3^ [3]

Pmet (beats min^−1^ kg^−1^) = (HR − 35) min^−1^ kg^−1^ [6]

Each pace was determined according to speed: walk (<1.67 m/s), trot (1.94–4.17 m/s) and gallop (>4.44 m/s).

Blood sampling and lactate analysis: Blood samples were taken from the jugular vein of each horse at T0 (at rest, before exercise), T1 (immediately after the first race), T2 (immediately after the second race), T3 (immediately after the third race), T4 (at 30 min of recovery) and T5 (at 240 min of recovery), into test tubes containing EDTA-sodium fluoride (Vacutainer, BD, São Paulo, Brazil) for measurement of plasma lactate concentration. The tubes were centrifuged using an 80-2B Centrifuge (Daiki, São Paulo, Brazil) and the refrigerated plasma was transported to the laboratory using a cooler with ice. Lactate concentration was determined using commercial kits (Bioclin, Belo Horizonte, Brazil) in an automatic biochemical analyser (PKL 125, Paramedical SRL, Salerno, Italy).

Statistical analysis: Results were analysed using a computerized statistical program (Minitab^®^ 19 Statistical Software, Lean SixSigma, Philadelphia, PA, USA) and were presented as mean ± SD. The normality of variables was checked with Kolmogorov–Smirnov test. The influence of exercise was evaluated through analysis of variance for repeated measures (one-way ANOVA), followed by comparison between means (Tukey test) for energy expenditure (EE), net cost transport (COT) and metabolic power (Pmet). Significance was set at *p* ≤ 0.05 in all cases.

## 3. Results

Significant differences were observed between PH and HH during the VST (Table 1, Table 2 and Table 3) for HR, EE, COT and Pmet. Significantly higher values of EE and HR were observed for HH while trotting in all three races of the VST, while PH had significantly higher values of HR, EE and COT when galloping in two of the three races. When considering total VST (three races), PH showed significant higher values for EE and COT and HH for Pmet (Table 2). Significantly high speed was observed for HH in just one race while walking and, oppositely, for PH in one race while galloping.

A significant effect of exercise (VST) was observed in PH and HH for plasma lactate, with higher values observed for the former ones (Table 4).

The equestrian course (~120 m) was performed in 40–50 s. According to riders, no signs of discomfort or reduced performance were observed during exercise execution.

## 4. Discussion

The results of this research show that the effort of each athlete is greater, for a given moment, in the sport, with the beginning being costlier for the HH, which has the role of aligning the bull, and the end is costlier for the PH, which has the role of maintaining acceleration and promoting the bull’s displacement, with its rider hanging on one side of the saddle.

The hypothesis proposed by the authors regarding the energy expenditure (EE) of PH was confirmed. As shown in Table 1 and Table 2, EE was significantly higher in PH during gallop and in exercise as a whole when comparing with values recorded for HH. EE was calculated indirectly using HR [1,3,6]. As shown in Table 3, HR was significantly higher in PH during gallop. PHs have the role of pulling the bull, causing it to fall and, thus, a higher HR and EE is expected. Moreover, this occurs at the peak of the gallop, which has a short duration (<60 s). The highest HR in PH was also described by Hunka et al. [16] and Sodre et al. [21]. According to these authors, HH have higher %HRs <150 beats/min (61% vs. 39% of PH), possibly indicating that their effort has a large aerobic contribution during VST than PH [10].

The higher EE of PH is also reflected in plasma lactate concentrations. Both categories of athletes worked with lactate indices above the anaerobic threshold of 4 mmol/L [25], which characterizes a predominance of the glycolytic energy production pathway in this type of effort (high-intensity and short-duration exercises) [16], similarly to other sports using Quarter Horses [26,27,28]. PH mobilized this energy pathway more, which has been also observed by other authors studying vaquejada [15,20,21], which corroborates the findings for HR. A higher lactate concentration was also associated with higher energy expenditure in Marchador horses [13], although these values were quite inferior in marcha gait [29].

In addition to a higher EE in PH, COT was also significantly higher in these animals during the VST. COT is usually used to estimate biomechanical efficiency during locomotion, defining the costs to move a unit of weight for a certain distance [3,12]. Although previous research has shown that gaits with lower speeds but shorter strides and greater stride frequency could lead to a greater lactate concentration, as well as greater energy requirements [3,13,30], the displacement of the bull in the moment of maximal acceleration, associated with the rider displacement on the saddle (to the opposite side of the bull), generated a mechanical/muscular overload, causing COT to be higher for PH even though both categories of athletes worked at the same speed within the same distance. According to Piccione et al. [12], COT is clearly dependent on weight.

In an unexpected but justified way, HH showed higher values for HR and EE while trotting, indicating greater energy depletion and greater physical effort in these animals at this stage of this sport’s equestrian modality. At this moment in the exercise, HH must flank the bull and lead it to the PH, and there are constant changes in its direction and speed (accelerations/decelerations). Thereby, Pmet was higher in VST for HH. Such findings are crucial; it is known that training sessions generate energy demands directly related to volume (quantitative component) and intensity (qualitative component) of exercise [10].

The results of the present research reinforce that PH and HH are different athletes and, therefore, that they also require different athletic conditioning and nutritional energy-management programs. The characterization of energy demands in equestrian sports have already been done [5,13]. Manso Filho et al. [5] observed that vaquejada horses spent much more energy (~51.42 mL O_2_/kg/min) than marcha (~34.9 mL O_2_/kg/min) or riding (~37.4 mL O_2_/kg/min) horses. All authors highlighted the importance of such results in evaluating physical conditioning and properly planning training schedules, considering the breed and competition in which horses will participate, to avoid generalizations [10,13].

The recovery of HR and plasma lactate values to pre-exercise levels (in stable) within 30 min of recovery denotes the good athletic conditioning of all horses used in the present experimental protocol [20]. This was observed for both PH and HH.

## 5. Conclusions

Results showed that PH had higher EE and COT, while HH had higher Pmet in a VST. However, during the trotting phase of exercise, HH spent more energy, with higher values of HR and EE. Despite practicing the same sport, PH and HH are distinct athletes, and these must be considered to set up appropriate physical and nutritional programs, leading to better performance and guarantee of well-being.

## Figures and Tables

**Table 1:** Energy expenditure (EE), cost of transport (COT) and metabolic power (Pmet) of each pace (walk, trot and gallop) in pull and helper horses after a VST.

Pace	Pull Horses	Helper Horses	*p*
	Energy Expenditure (J/kg/min)		
Walk			
1st race	74.05 (±1.49)	71.48 (±1.33)	0.817
2nd race	75.79 (±1.36)	75.96 (±1.28)	0.914
3rd race	72.66 (±1.42)	73.88 (±1.31)	0.898
Trot			
1st race	92.59 (±1.35) ^b^	130.43 (±1.38) ^a^	0.010
2nd race	149.33 (±1.48) ^b^	165.39 (±1.40) ^a^	0.002
3rd race	93.68 (±1.35) ^b^	131.13 (±1.36) ^a^	0.001
Gallop			
1st race	203.49 (±1.36) ^a^	184.17 (±1.32) ^b^	0.000
2nd race	189.14 (±1.44)	195.43 (±1.27)	0.762
3rd race	194.98 (±26) ^a^	179.30 (±1.29) ^b^	0.010
	Cost of transport (beats/kg/m × 10^3^)		
Walk			
1st race	27.65 (±1.15)	28.93 (±1.24)	0.912
2nd race	59.59 (±1.10)	47.48 (±1.11)	0.766
3rd race	27.01 (±1.22)	37.10 (±0.59)	0.645
Trot			
1st race	33.10 (±1.27)	42.05 (±1.27)	0.745
2nd race	54.56 (±1.33)	43.63 (±1.22)	0.876
3rd race	37.38 (±1.25)	46.47 (±1.17)	0.882
Gallop			
1st race	46.65 (±1.23)^a^	29.66 (±1.18)^b^	0.000
2nd race	45.41 (±1.25)	39.26 (±1.21)	0.065
3rd race	46.40 (±1.23)^a^	30.90 (±1.20)^b^	0.001
	Metabolic Power (beats/min/kg)		
Walk			
1st race	117.00 (±1.14) ^b^	153.70 (±1.18) ^a^	0.000
2nd race	200.10 (±1.41)	204.40 (±1.11)	0.982
3rd race	205.20 (±1.12)	298.20 (±1.23)	0.881
Trot			
1st race	182.10 (±1.10) ^b^	251.30 (±1.23) ^a^	0.002
2nd race	249.10 (±1.24)	223.40 (±0.94)	0.829
3rd race	192.30 (±1.26) ^b^	442.80 (±1.11) ^a^	0.000
Gallop			
1st race	263.70 (±1.19)	240.80 (±1.09)	0.922
2nd race	209.20 (±0.77)	222.55 (±1.05)	0.916
3rd race	266.90 (±1.07) ^b^	318.20 (±1.10) ^a^	0.002

Data expressed as mean values and standard deviation. Different letters in the same line denote significant differences (*p* ≤ 0.05).

**Table 2:** Total energy expenditure (EE), cost of transport (COT) and metabolic power (Pmet) in pull and helper horses after a VST.

Parameter	Pull Horses	Helper Horses	*p*
Energy Expenditure (J/kg/min)	150.59 (±1.67) ^a^	140.43 (±1.57) ^b^	0.027
Cost of transport (beats/kg/m × 10^3^)	42.86 (±1.36) ^a^	36.39 (±1.21) ^b^	0.003
Metabolic Power (beats/min/kg)	207.04 (±1.13) ^b^	264.15 (±1.18) ^a^	0.000

Data expressed as mean values and standard deviation. Different letters in the same line denote significant differences (*p* ≤ 0.05).

**Table 3:** Heart rate and velocity of each pace (walk, trot and gallop) in pull and helper horses after a VST.

Pace	Pull Horses	Helper Horses	*p*
	Heart Rate (beats/min)		
Walk			
1st race	91.33 (±1.24) ^b^	111.27 (±1.16) ^a^	0.002
2nd race	138.17 (±1.22)	136.78 (±1.36)	0.854
3rd race	86.61 (±1.12) ^b^	115.69 (±1.27) ^a^	0.000
Trot			
1st race	125.67 (±1.24) ^b^	148.59 (±1.21) ^a^	0.001
2nd race	154.44 (±1.64) ^b^	170.44 (±1.24) ^a^	0.011
3rd race	126.55 (±1.20) ^b^	150.04 (±1.26) ^a^	0.000
Gallop			
1st race	189.98 (±1.13) ^a^	178.54 (±1.15) ^b^	0.000
2nd race	181.22 (±1.20)	184.10 (±1.13)	0.415
3rd race	184.51(±1.18) ^a^	174.70 (±1.21) ^b^	0.008
	Velocity (m/s)		
Walk			
1st race	0.77 (±0.46) ^b^	1.10 (±0.47) ^a^	0.035
2nd race	1.18 (±0.46)	1.24 (±0.37)	0.309
3rd race	0.95 (±0.51)	1.21 (±0.48)	0.124
Trot			
1st race	3.30 (±0.81)	3.18 (±0.57)	0.630
2nd race	3.05 (±0.58)	2.80 (±0.65)	0.252
3rd race	3.02 (±0.64)	2.66 (±0.60)	0.093
Gallop			
1st race	6.95 (±0.41) ^a^	6.28 (±0.49) ^b^	0.003
2nd race	6.97 (±0.33)	7.27 (±0.52)	0.197
3rd race	6.71 (±0.36) ^a^	6.39 (±0.42)	0.134

Data expressed as mean values and standard deviation. Different letters in the same line denote significant differences (*p* ≤ 0.05).

**Table 4:** Plasma lactate measured in pull and helper horses, after a VST.

	Experimental Period						*p*	
	T0	T1	T2	T3	T4	T5	Physical Activity	Animal Category
PH	2.2 ± 0.5	8.0 ± 2.2	9.5 ± 2.7	10.6 ± 2.6	4.1 ± 2.3	1.8 ± 0.6	<0.0001	0.0433
HH	2.1 ± 1.1	5.6 ± 1.6	6.3 ± 2.1	6.7 ± 2.9	2.3 ± 0.4	2.3 ± 0.8	<0.0001	

Data expressed as mean values and standard deviation. Different letters in the same line denote significant differences by Tukey test (*p* ≤ 0.05). T0: at rest; T1: immediately after the first race; T2: immediately after the second race; T3, immediately after the third race; T4, at 30 min of recovery; T5, at 4 h of recovery. PH, pull horses; HH, helper horses.

## Data Availability

The data that support the findings of this study are not publicly available as it contains information that could compromise the privacy of research participants.
